# *In-Line* Monitoring of Polyhydroxyalkanoate (PHA) Production during High-Cell-Density Plant Oil Cultivations Using Photon Density Wave Spectroscopy

**DOI:** 10.3390/bioengineering6030085

**Published:** 2019-09-19

**Authors:** Björn Gutschmann, Thomas Schiewe, Manon T.H. Weiske, Peter Neubauer, Roland Hass, Sebastian L. Riedel

**Affiliations:** 1 Bioprocess Engineering, Department of Biotechnology, Technische Universität Berlin, 13355 Berlin, Germany; bjoern.gutschmann@tu-berlin.de (B.G.); peter.neubauer@tu-berlin.de (P.N.); 2 innoFSPEC, University of Potsdam, 14476 Potsdam, Germany; tschiewe@uni-potsdam.de (T.S.); rh@pdw-analytics.de (R.H.)

**Keywords:** polyhydroxyalkanoate, PHA, process analytical technologies, PAT, plant oil, high-cell-density fed-batch, photon density wave spectroscopy, PDW, *Ralstonia eutropha*, *Cupriavidus necator*, *on-line*, *in-line*

## Abstract

Polyhydroxyalkanoates (PHAs) are biodegradable plastic-like materials with versatile properties. Plant oils are excellent carbon sources for a cost-effective PHA production, due to their high carbon content, large availability, and comparatively low prices. Additionally, efficient process development and control is required for competitive PHA production, which can be facilitated by *on-line* or *in-line* monitoring devices. To this end, we have evaluated photon density wave (PDW) spectroscopy as a new process analytical technology for *Ralstonia eutropha* (*Cupriavidus necator*) H16 plant oil cultivations producing polyhydroxybutyrate (PHB) as an intracellular polymer. PDW spectroscopy was used for *in-line* recording of the reduced scattering coefficient *µ*_s_’ and the absorption coefficient *µ*_a_ at 638 nm. A correlation of *µ*_s_’ with the cell dry weight (CDW) and *µ*_a_ with the residual cell dry weight (RCDW) was observed during growth, PHB accumulation, and PHB degradation phases in batch and pulse feed cultivations. The correlation was used to predict CDW, RCDW, and PHB formation in a high-cell-density fed-batch cultivation with a productivity of 1.65 g_PHB_·L^−1^·h^−1^ and a final biomass of 106 g·L^−1^ containing 73 wt% PHB. The new method applied in this study allows *in-line* monitoring of CDW, RCDW, and PHA formation.

## 1. Introduction

When the US Food and Drug Administration (FDA) announced their process analytical technology (PAT) directives, the investigation of PAT became a key research area in bioprocess development. The main objectives are designing, developing, and operating bioprocesses to guarantee a targeted final product quality [1,2]. The focus of this initiative was predominantly on biopharmaceutical processes, while novel PAT tools could be integrated into any bioprocess. Especially, the implementation of PAT for polyhydroxyalkanoate (PHA) production can provide significant benefits to facilitate a consistent and highly efficient production. Techniques such as FTIR, Raman spectroscopy, fluorescence staining associated with flow cytometry, and enzymatic approaches were reported as novel methods for a rapid characterization of PHA production [3,4,5,6,7]. A comprehensive overview of qualitative and quantitative methods for PHA analysis was published by Koller et al. [8]. However, the reported methods have not been applied for *in-line* or *at-line* measurements of the PHA production process so far.

Photon density wave (PDW) spectroscopy is an *in-line* technique, which has been used as an analytical tool for measurements of various highly turbid chemical processes [9,10,11,12]. The method is based on the theory of photon migration in multiple light scattering material. If intensity-modulated light is introduced into a strongly light scattering but weakly light absorbing material, a PDW is generated. Absorption and scattering properties of the material influence the amplitude and phase of the PDW. By quantifying these shifts as a function of the emitter fiber and detector fiber distance and of the modulation frequency, the absorption coefficient *µ*_a_ and the reduced scattering coefficient *µ*_s_’ can be determined independently [9,13,14]. The mentioned features make PDW spectroscopy very attractive for the monitoring of high-cell-density bioprocesses.

Currently, PHA production costs are not compatible with the low-priced production of conventional plastics. The main cost driving factors are the feedstocks for PHA accumulation and the recovery process. Thus, alternative low-cost substrates, e.g., biogenic waste streams, are of high interest to reduce the final production price. Other attempts concentrate on finding more sustainable and price efficient purification strategies [15,16,17,18,19,20,21]. *Ralstonia eutropha* (also known as *Cupriavidus necator*) is one of the main species studied for polyhydroxybutyrate (PHB) accumulation and the model organism for PHA accumulation [22]. Growth of *R. eutropha* on oleaginous feedstocks is particularly attractive due to their high carbon contents, high conversion rates to PHA, and low culture dilution in fed-batch processes. Efficient growth on these feedstocks is facilitated by the expression of extracellular lipases, which emulsify the lipids [23,24,25,26,27]. A large biomass accumulation prior to PHA accumulation is very important for a high final product titer. In this context, it has been shown that urea is an inexpensive nitrogen source, which allows excellent growth [24,28]. Despite alternative substrates and downstream approaches, highly efficient bioprocesses are required for an economic feasible PHA production. Recently, high-cell-density cultivations with *R. eutropha* on various renewable feedstocks have been published presenting the production of over 100 g·L^−1^ PHA and space time yields from 1 to 2.5 g_PHA_·L^−1^·h^−1^ [21,24,29,30,31]. However, none of the presented studies describe *in-line* PAT-based monitoring or control strategies for the enhancement of process results.

This work aims to integrate PDW spectroscopy into high-cell-density bioprocesses, for the monitoring of the highly turbid and complex PHB production with *R. eutropha* in plant oil cultivations. As a result, total cell dry weight (CDW) and residual cell dry weight (RCDW, the difference of CDW and the PHB concentration) accumulation could be distinguished with the PDW spectroscopy probe as a new *in-line* tool for bioprocesses.

## 2. Materials and Methods 

### 2.1. Bacterial Strain

All cultivations were performed with the wild type strain *R. eutropha* H16 (DSM-428, Leibniz Institute DSMZ-German Collection of Microorganisms and Cell Cultures, Germany). 

### 2.2. Growth Media and Preculture Cultivation Conditions 

Tryptic soy broth (TSB) media (17 g·L^−1^ tryptone, 5 g·L^−1^ NaCl, 3 g·L^−1^ peptone) was used for the first precultures and with an additional supply of 2% (w·v^−1^) agar for culture plates. The second precultures and bioreactor cultivations were conducted in mineral salt media (MSM) containing 4.62 g·L^−1^ NaH_2_PO_4_·H_2_O, 5.74 g·L^−1^ Na_2_HPO_4_·2H_2_O, 0.45 g·L^−1^ K_2_SO_4_, 0.04 g·L^−1^ NaOH, 0.80 g·L^−1^ MgSO_4_·7H_2_O, 0.06 g·L^−1^ CaCl_2_·2H_2_O and 1 mL·L^−1^ trace element solution consisting of 0.48 g·L^−1^ CuSO_4_·5H_2_O, 2.4 g·L^−1^ ZnSO_4_·7H_2_O, 2.4 g·L^−1^ MnSO_4_·H_2_O, 15 g·L^−1^ FeSO_4_·7H_2_O. All cultivation media and plates contained 10 mg·L^−1^ sterile filtered gentamycin sulfate. Rapeseed oil (Edeka Zentrale AG & Co. KG, Germany) was used as the sole carbon source and urea as the sole nitrogen source in the MSM. The explicit amounts are described in the text. All chemicals were purchased from Carl Roth GmbH & Co. KG (Germany) unless stated otherwise.

*R. eutropha* H16 was streaked from a cryoculture on a TSB agar plate and incubated for 3–4 days at 30 °C. A single colony from the plate was used to inoculate the first preculture in 10 mL TSB media in a 125-mL Ultra Yield™ Flask (Thomson Instrument Company, USA) sealed with an AirOtop™ enhanced flask seal (Thomson Instrument Company, USA). After incubating for 16 h, 2.5 mL were used to inoculate the second preculture (250 mL MSM with 3% (w·v^−1^) rapeseed oil and 4.5 g·L^−1^ urea) in a 1-L DURAN^®^ baffled glass flask with a GL45 thread (DWK Life Sciences GmbH, Germany) sealed with an AirOtop membrane. After 24 h of incubation, the complete second preculture was used to inoculate the main bioreactor culture. The precultures were incubated at 30 °C and shaken at 200 rpm (first preculture) or 180 rpm (second preculture) in an orbital shaker (Kühner LT-X incubator, Adolf Kühner AG, Switzerland, 50 mm amplitude). 

### 2.3. Bioreactor Cultivation Conditions

Mineral salts dissolved in deionized (DI) water and rapeseed oil were added prior autoclavation in a 6.6-L stirred tank bioreactor with two six-blade Rushton impellers (BIOSTAT^®^ Aplus, Sartorius AG, Germany). MgSO_4_, CaCl_2_, trace elements, gentamycin, and urea were added into the medium after autoclavation from sterile stock solutions. The temperature was maintained at 30 °C and the pH was kept constant at 6.8 ± 0.2 using 2 M NaOH and 1 M H_3_PO_4_ for pH control. The dissolved oxygen concentration (DO) was kept above 40% using a stirrer cascade ranging from 400 to 1350 rpm. The cultures were aerated with a constant aeration rate of 0.5 vvm throughout the cultivations. Five pairs of cable ties were mounted on the upper part of the stirrer shaft in order to break the foam mechanically and thus preventing overfoaming of the reactor.

#### 2.3.1. Batch Cultivations

For a first evaluation of the PDW spectroscopy signal, three batch cultivations were performed in which the carbon and nitrogen content was varied. The concentrations of rapeseed oil were 3, 4, and 4% (w·v^−1^), and 2.25 (corresponding to 75 mM nitrogen), 4.5, and 2.25 g·L^−1^ for urea, respectively.

#### 2.3.2. Pulse-Based Fed-Batch Cultivation

A cultivation strategy with a pulse feeding was performed in biological duplicates. The cultures initially contained 0.5% (w·v^−1^) rapeseed oil and 4.5 g·L^−1^ urea. Pulses were given whenever the PDW spectroscopy *in-line* signal (*µ*_s_’ at 638 nm) indicated a decreased cell activity. After 8.2 h, the first pulse (15 g rapeseed oil) was added, followed by two more rapeseed oil pulses at 14.3 h (30 g) and at 21.1 h (60 g). At 31.7 h a pulse consisting of 110 mL urea solution (122 g·L^−1^), 15.6 mL 0.5 M K_2_SO_4_, 30 mL 0.042 M CaCl_2_, 30 mL 0.32 M MgSO_4_, and 3 mL trace element solution was added to restore the initial media concentrations of the components. The last pulse (120 g rapeseed oil) was added after 48.4 h.

#### 2.3.3. Fed-Batch High-Cell-Density Cultivation

The culture initially contained 4% (w·v^−1^) rapeseed oil and 4.5 g·L^−1^ urea (150 mM nitrogen). Continuous feeding of pure rapeseed oil and a 30% (w·v^−1^) urea solution was started 7 h after inoculation with initial feeding rates of 3.5 g·h^−1^ and 0.39 mL·h^−1^, respectively. Both feeding rates were linearly increased up to 6.58 mL·h^−1^ (urea) at 16 h, after which the urea feed was stopped to cause nitrogen starvation, and 23 g·h^−1^ (rapeseed oil) at 35 h to final concentrations of 480 mM nitrogen and 170 g·L^−1^ rapeseed oil. A single injection of MgSO_4_, CaCl_2_, K_2_SO_4_, and trace elements was performed after 20 h with the amounts as described above (see Section 2.3.2.) to restore the initial concentrations and prevent nutrient depletion. An additional pulse of CaCl_2_ with the same concentration was added after 32 h. 

### 2.4. Photon Density Wave Spectroscopy

**Figure 1:**
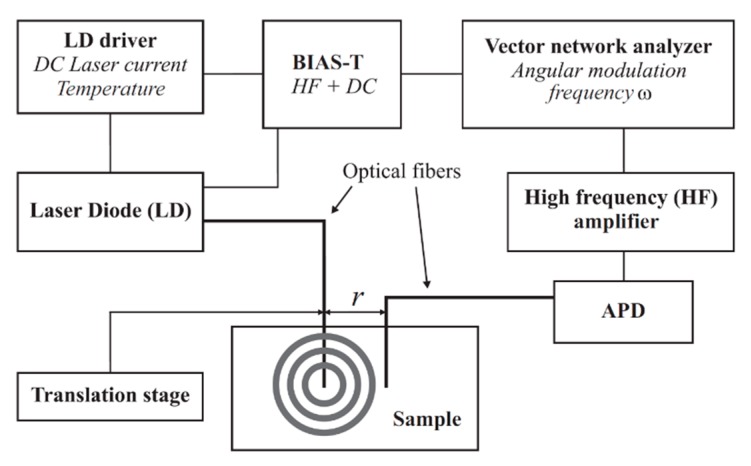
Schematic experimental set-up of a photon density wave (PDW) spectrometer [13].

A PDW spectrometer built by the University of Potsdam was used for the measurement of the absorption coefficient *µ*_a_ and the reduced scattering coefficient *µ*_s_’. Identical devices are commercially available at PDW Analytics GmbH (Potsdam, Germany). The general set-up of the PDW spectrometer was described by Bressel et al., as follows: “A schematic set-up of the spectrometer is shown in Figure 1. Light from a laser diode with wavelength λ [m] (typ. 400–1000 nm) is sinusoidally intensity modulated by a vector network analyzer (typ. f = ω/(2π) = 10–1300 MHz). The light is then coupled into the material via an optical fiber, acting as a point-like light source. A second optical fiber, positioned at a distance r to the emission fiber (typ. r = 5–30 mm), collects light of the PDW and guides it onto an avalanche photodiode (APD) as detector.” [13]. 

To integrate the multifiber PDW spectroscopy *in-line* probe into the system, a DN25 safety Ingold socket (elpotech GmbH & Co. KG, Germany) was welded onto the lid of the bioreactor. The probe was mounted before autoclaving and sterilized with the bioreactor inside the autoclave. The optical fibers of the probe were connected to the PDW spectrometer after autoclaving. *µ*_s_’ and *µ*_a_ were analyzed at 638 nm with a temporal resolution of 0.8 min^−1^. A 10-point moving average was used to reduce the signal noise. 

### 2.5. Analytical Methods

For each *off-line* reference analysis time point, two aliquots of 10 mL were sampled in preweighed 15-mL polypropylene test tubes. The samples were centrifuged for 15 min at 6000× *g* and pellets were washed either with a mixture of 5 mL cold deionized (DI) water and 2 mL cold hexane or with 7 mL cold DI water to remove residual lipids. The washed pellets were resuspended in 2–4 mL ice cold DI water, frozen at −80 °C, and dried for 24 h by lyophilization (Gamma 1–20, Martin Christ Gefriertrocknungsanlagen GmbH, Germany). Then the CDW was determined by weighing the test tubes. 

The PHB content was determined *off-line* by high-performance liquid chromatography with a diode array detector (HPLC-DAD 1200 series, Agilent Technologies, USA). The method was adapted from Karr et al. [32]. Pure PHB (Sigma-Aldrich Corporation, USA) or 8–15 mg freeze-dried cells were depolymerized by boiling samples with 1 mL concentrated H_2_SO_4_ to yield crotonic acid. Dilution series of the depolymerized PHB were prepared to yield standards in the range of 0.1–10 mg·mL^−1^. Samples were diluted with 4 mL 5 mM H_2_SO_4_, filtered through a 0.2 µm cellulose acetate syringe filter, and subsequently 100 µL were transferred to a HPLC vial containing 900 µL of 5 mM H_2_SO_4_. HPLC analysis was performed with an injection volume of 20 µL using 5 mM H_2_SO_4_ as an eluent with an isocratic flow rate of 0.4 mL·min^−1^ for 60 min on a NUCLEOGEL^®^ ION 300 OA column (Macherey-Nagel, Germany). Crotonic acid was detected at 210 nm. RCDW was determined by subtracting the PHB from the CDW concentration.

The nitrogen content was indirectly determined *off-line* by measuring ammonia, resulting from urea cleavage, in the supernatant using a pipetting robot (Cedex Bio HT Analyzer, Roche Diagnostics International AG, Switzerland) with the NH3 Bio HT test kit (Roche Diagnostics International AG, Switzerland).

During the fed-batch cultivation the *in-line* PDW spectroscopy signals were used for an *on-line* determination of the CDW, RCDW, and PHB content (see Section 3.4). 

## 3. Results

A reduction of the PHA production price is crucial for commercialization [33], which can be facilitated by maximization of the biotechnological process performance using PAT. In this context, our group is interested in developing biotechnological processes for PHA production from renewable resources [24] and biogenic waste streams [17,18].

In this study, PDW spectroscopy was evaluated as a novel *in-line* tool to monitor the PHA production process with *R. eutropha* from plant oil. 

### 3.1. Batch Cultivations

**Figure 2:**
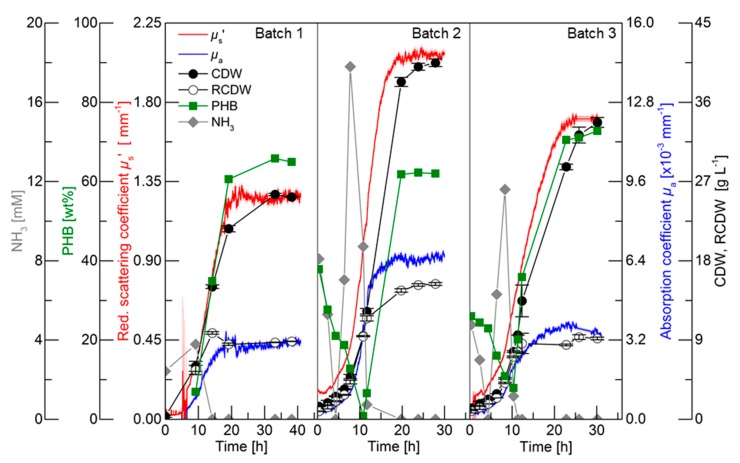
Batch cultivations for polyhydroxybutyrate (PHB) production by *R. eutropha* H16. Batch 1 contained 3% (w·v^−1^) rapeseed oil and 2.25 g·L^−1^ urea (75 mM nitrogen), Batch 2 contained 4% (w·v^−1^) rapeseed oil and 4.5 g·L^−1^ urea, and Batch 3 contained 4% (w·v^−1^) rapeseed oil and 2.25 g·L^−1^ urea. Ammonia content (grey diamonds, mM), PHB content (green squares, wt%), cell dry weight (CDW) (filled circles, g·L^−1^), residual cell dry weight (RCDW) (empty circles, g·L^−1^), reduced scattering coefficient *µ*_s_’ (red line, mm^−1^), and absorption coefficient *µ*_a_ (blue line, ×10^−3^ mm^−1^) at 638 nm are shown. Error bars indicate minimum and maximum values of technical duplicates.

For an initial evaluation of the PDW spectroscopy signals, batch cultivations with different C/N ratios were performed to trigger different PHB and CDW accumulation. The results of the three batch cultivations are shown in Figure 2. Detailed graphs of the cultivations can be found in the in Supplementary Materials in Figures S1–S3. The first batch cultivation was performed as a reference batch containing 3% (w·v^−1^) rapeseed oil and 75 mM nitrogen (2.25 g·L^−1^ urea). Nitrogen limitation was indirectly detected by ammonia quantification, which is released from urea cleavage prior nitrogen uptake [24,28]. Nitrogen was depleted between 9.2 and 14.2 h, which triggered PHB accumulation. Within this period, the CDW increased from 6 to 15 g·L^−1^ and the PHB content increased from 7 to 35 wt% (0.4 to 5.3 g_PHB_·L^−1^). The PDW spectroscopy signals *µ*_a_ and *µ*_s_’ did not show any analyzable signals until 6.5 h. Subsequently, both signals exponentially increased until 10.5 h. After this time point, *µ*_a_ linearly increased until 16 h and did not further rise afterwards. An increase of the *µ*_s_’ signal was detected from 10.5 to 20 h and remained constant afterwards. The maximum CDW (25.5 g·L^−1^) was achieved after 33.2 h containing 65 wt% PHB (16.8 g_PHB_·L^−1^), which represents an overall yield of 0.56 g_PHB_·g_Oil_^−1^. Over the entire cultivation, the urea consumption for biomass accumulation was 0.26 g_Urea_·g_RCDW_^−1^.

The purpose of Batch 2 was to decrease the C/N ratio compared to the reference batch for an increased accumulation of active biomass (RCDW) and decreased PHB content. Nitrogen was depleted between 11.6 and 19.8 h. Within this period, the CDW and PHB content increased from 12.3 to 38.3 g·L^−1^ and 7 to 62 wt% (0.8 to 23.7 g_PHB_·L^−1^), respectively. The PDW spectroscopy signals *µ*_a_ and *µ*_s_’ simultaneously increased from the beginning of the cultivation until 12 h in an exponential manner. No significant changes of *µ*_a_ were detected after that time point, whereas *µ*_s_’ increased until 18 h and subsequently remained constant. After 23.8 h the maximum CDW of 40 g·L^−1^ containing 63 wt% (26.2 g_PHB_·L^−1^) was achieved. Overall, a PHB yield of 0.66 g_PHB_·g_Oil_^−1^ and urea consumption of 0.29 g_Urea_·g_RCDW_^−1^ was achieved.

The C/N ratio in the third batch was increased compared to the reference batch by keeping the initial nitrogen concentration constant (75 mM) but increasing the rapeseed oil content to 4% (w·v^−1^). Depletion of nitrogen occurred between 10.3 and 11.3 h. Within this period the CDW increased from 7.7 g·L^−1^ with 8 wt% PHB (0.6 g_PHB_·L^−1^) to 9.6 g·L^−1^ with 20 wt% PHB (1.9 g_PHB_·L^−1^). PDW signals were detectable after 2 h. A comparable increase of *µ*_a_ and *µ*_s_’ was detected until 10 h. An attenuated increase of *µ*_a_ until 17.5 h was detected, whereas a diminished increase of *µ*_s_’ was detected from 10 to 22.5 h. The CDW further increased to 33.7 g·L^−1^ containing 73 wt% PHB (24.5 g_PHB_·L^−1^). Overall, a PHB yield of 0.61 g_PHB_·g_Oil_^−1^ and urea consumption of 0.25 g_Urea_·g_RCDW_^−1^ was achieved.

To summarize, the C/N ratio influenced the yield coefficients for PHB accumulation and urea usage within these three batch cultivations. During the batch cultivations, *µ*_s_’ and *µ*_a_ simultaneously increased until nitrogen depletion. Subsequently, *µ*_a_ leveled off and *µ*_s_’ increased further until maximum PHB accumulation.

### 3.2. Pulse-Based Fed-Batch Cultivation

**Figure 3:**
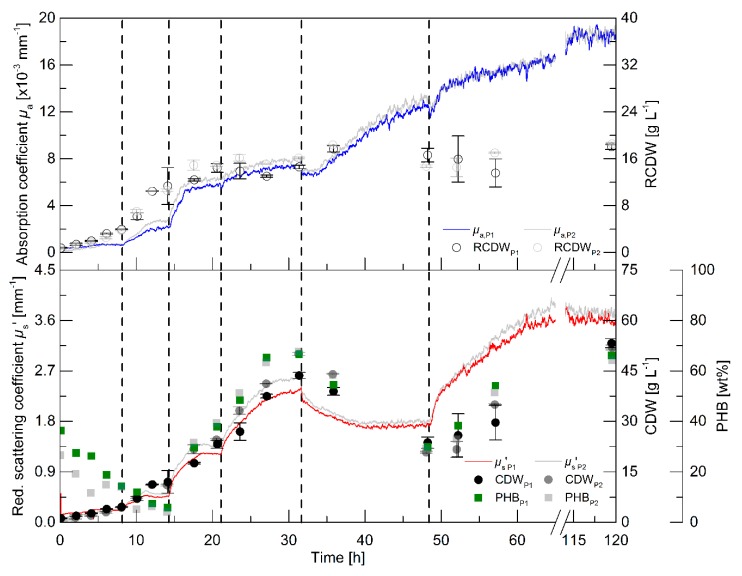
Pulse feeding cultivations for PHB production by *R. eutropha* H16. The cultures were initially started with 0.5% (w·v^−1^) rapeseed oil and 4.5 g·L^−1^ urea (150 mM nitrogen). The dashed vertical lines represent time points of pulse additions: 0.5% (w·v^−1^) rapeseed oil at 8.2 h, 1% (w·v^−1^) rapeseed oil at 14.3 h, and 2% (w·v^−1^) rapeseed oil at 21.1 h. At 31.7 h a bolus consisting of 110 mL urea solution (122 g·L^−1^), 15.6 mL 0.5 M K_2_SO_4_, 30 mL 0.32 M MgSO_4_, 30 mL 0.042 mM CaCl_2_, and 3 mL trace element solution was added to the bioreactor. A final bolus of 4% (w·v^−1^) rapeseed oil was added at 48.4 h. Data from the reference experiment is shown in color (indexed P1) and the biological duplicate (with an independent seed train) in transparent grey (indexed P2). Absorption coefficient *µ*_a_ (upper graph, solid line, ×10^−3^ mm^−1^), RCDW (upper graph, empty circles, g·L^−1^), PHB content (bottom graph, squares, wt%), CDW (bottom graph, filled circles, g·L^−1^), and reduced scattering coefficient *µ*_s_’ (bottom graph, solid line, mm^−1^) are shown. Error bars indicate minimum and maximum values of technical duplicates.

While the batch cultivations aimed to initially evaluate the relationship of the PDW spectroscopy signals with process relevant characteristics, a pulse-based fed-batch cultivation was conducted to: (i) show the feasibility to control the process by monitoring the process with PDW spectroscopy; (ii) confirm signal relationships; and (iii) validate the reproducibility during biological duplicate cultivations with independent seed trains. The process intention was implemented without difficulty: a pulse of either rapeseed oil or a nutrient bolus (see dashed lines in Figure 3 and details in the legend), respectively, was added to the culture when the *µ*_s_’ signal showed no further changes and indicated either carbon or nitrogen limitation. It is worth mentioning, that no signal deflections were observed whenever oil pulses were added to the bioreactor, which would account for an effect of the added oil on *µ*_s_’ or *µ*_a_.

Within the first 8 h, the intracellular PHB content decreased from 36 to 14 wt% (Figure 3). An increase of the CDW, RCDW, *µ*_s_’ and *µ*_a_ was observed in the first 6 h of the cultivation. Subsequently, only minor increases of the CDW and RCDW of about 0.7 g·L^−1^ were detected until 8 h, whereas the PDW spectroscopy signals did not further increase during this period. After addition of the first pulse (0.5% (w·v^−1^) rapeseed oil), *µ*_s_’ and *µ*_a_ resumed to increase until reaching constant levels at 11.5 h until the next pulse addition (1% (w·v^−1^) rapeseed oil). The increase of *µ*_a_ stopped at 16.5 h, whereas *µ*_s_’ increased further until 18 h. Nitrogen depletion was detected at 17.5 h (Figure S4, Supplementary Materials). The PHB content had already increased to 30 wt% at this time point and further increased to 38 wt% before addition of the next pulse. The CDW increased up to 23 g·L^−1^, whereas the RCDW stopped at a value of 14 g·L^−1^ at 20.5 h. Addition of the next rapeseed oil pulse (2% (w·v^−1^)) at 21 h did not trigger a significant change of the *µ*_s_’ signal and of the RCDW. In contrast, a sharp increase of *µ*_s_’ resulted from the rapeseed oil addition and the CDW increased up to 43 g·L^−1^ at 31 h containing 66 wt% PHB. At 31.6 h, a bolus containing urea as a nitrogen source was supplemented to the culture. The addition resulted in a dilution of the culture, which was seen in a step decrease of both PDW spectroscopy signals at this time point. Subsequently, *µ*_s_’ decreased until 42 h and stayed constant until the next pulse addition. In contrast, *µ*_a_ resumed to increase until 45 h. The CDW decreased during this period to 24 g·L^−1^, resulting from intracellular PHB degradation. The PHB content decreased to 30 wt%. At the same time, the RCDW increased to 17 g·L^−1^. The addition of the next pulse (4% (w·v^−1^) rapeseed oil) resulted in resumed growth on rapeseed oil as the primary carbon source instead of degrading the intracellular carbon storage. The PDW spectroscopy signals increased after the rapeseed oil supplementation. The scattering signal *µ*_s_’ increased until 70 h, whereas the absorption coefficient *µ*_a_ only slightly increased after 52 h. At 52 h, nitrogen was depleted again. The cells accumulated 66 wt% PHB until the end of the cultivation and the CDW increased to 53 g·L^−1^. When the whole cultivation was repeated, the PDW spectroscopy signals showed an equivalent course and a final CDW of 52 g·L^−1^ CDW containing 64 wt% PHB, indicating a high robustness, i.e., repeatability, of the process. 

The pulse-based fed-batch experiment showed the possibility to control the rapeseed oil-based cultivation for PHB production using the *in-line* PDW spectroscopy probe. The highly reproducible course of the PDW spectroscopy signals strongly imply the connection of biological events with this measurement technique. The coefficients *µ*_s_’ and *µ*_a_ show the same trend as the CDW and RCDW, respectively. Nevertheless, a more significant change was observed for the *µ*_a_ signal after addition of the nitrogen pulse than the *off-line* determined RCDW. Regardless of this discrepancy, the hypothesis was that the *µ*_s_’ and *µ*_a_ correspond and can be correlated with the CDW and RCDW, respectively.

### 3.3. PDW Spectroscopy Signal Correlation

**Figure 4:**
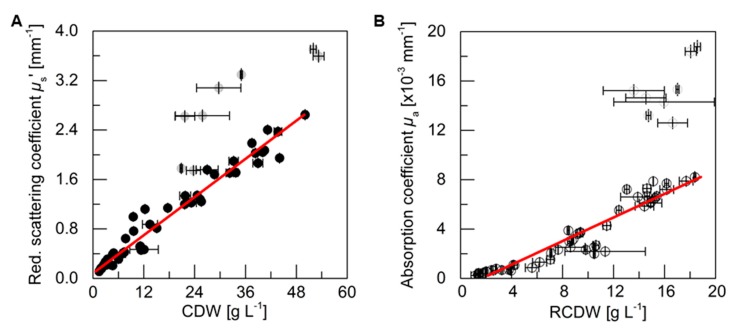
Correlation analysis of PDW spectroscopy signals with *off-line* values. (**A**) Reduced scattering coefficient *µ*_s_’ at 638 nm is correlated with CDW. (**B**) Absorption coefficient *µ*_a_ at 638 nm is correlated with RCDW. Data points are from five cultivations with different rapeseed oil and urea contents (cf. Figure 2 and Figure 3). The gray data points comprise the last four samples of the two pulse-based fed-batch experiments (cf. Figure 3), which differ significantly from the other samples and were therefore not considered for the linear fit with the experimental data. A squared correlation coefficient R^2^ of 0.96 was obtained for *µ*_s_’ and CDW and R^2^ of 0.90 for *µ*_a_ and RCDW. Error bars indicate minimum and maximum values of technical duplicates (CDW, RCDW). STDEV of the 10-point average is shown for *µ*_s_’ and *µ*_a_.

For an analysis of the correlation between the PDW spectroscopy signals *µ*_s_’ and *µ*_a_ at 638 nm, respectively, with process relevant characteristics, the experiments described above were analyzed. The reduced scattering coefficient *µ*_s_’ followed the course of the CDW, while the absorption coefficient *µ*_a_ increased with the rise of RCDW. The correlation of the respective values from all five cultivations (i.e., batch and pulse-based fed-batch cultures) are shown in Figure 4. A root mean squared error of 0.96 for the linear correlation of *µ*_s_’ and the CDW was obtained, whereas the linear correlation of *µ*_a_ and the RCDW resulted in a R^2^ of 0.90. Equations (A1)–(A3) in Appendix A show the obtained formulas for calculating the CDW, RCDW, and subsequently the PHB concentration using the linear relationships of the *in-line* PDW spectroscopy signals with the corresponding *off-line* values. Due to disproportionally large PDW spectroscopy signal intensification after 48 h of the pulse feeding experiments (Figure 3), the last four data points of each replicate were not included in the correlation analysis.

### 3.4. High-Cell-Density Fed-Batch Cultivation

**Figure 5:**
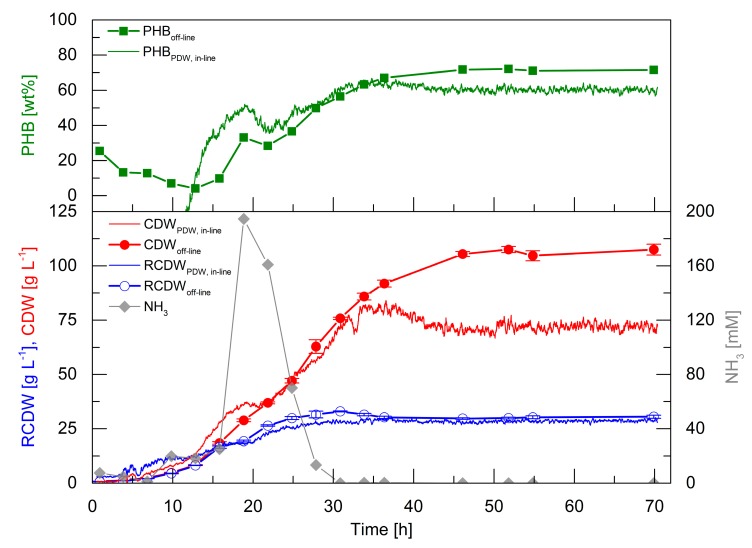
Fed-batch cultivation of *R. eutropha* H16 for PHB production. The culture was started with 4% (w·v^−1^) rapeseed oil and 4.5 g·L^−1^ urea (150 mM nitrogen). Rapeseed oil feeding linearly increased from 7 h with an initial feeding rate of 3.5 g·h^−1^ to 35 h with a final feeding rate of 23 g·h^−1^ up to a total concentration of 17% (w·v^−1^). Urea (30% (w·v^−1^)) feeding was linearly increased from 7 h with an initial feeding rate of 0.39 mL·h^−1^ up to a final feeding rate of 6.58 mL·h^−1^ at 16 h to a total concentration of 14.4 g·L^−1^. *In-line* PHB content (upper graph, green line, wt%) by PDW spectroscopy and *off-line* PHB content (upper graph, green squares, wt%), estimated *in-line* CDW by PDW spectroscopy (bottom graph, red line, g·L^−1^), *off-line* CDW (bottom graph, red filled circles, g·L^−1^), estimated *in-line* RCDW by PDW spectroscopy (bottom graph, blue line, g·L^−1^), *off-line* RCDW (bottom graph, blue empty circles, g·L^−1^), and ammonia content (bottom graph, grey diamonds, mM) are shown. Error bars indicate minimum and maximum values of technical duplicates.

High-cell-density cultivations are essential for a competitive production of PHA biopolymers. Therefore, it was aimed to perform such a cultivation to evaluate the PDW spectroscopy probe performance at industrial relevant biomass concentrations. The correlation factors obtained from the first five cultivations (Equations (A1)–(A3) in Appendix A) were used to calculate the CDW, RCDW, and PHB content from the *in-line* PDW spectroscopy signals (Figure 5). 

In the first 13 h, the *off-line* CDW increased to 8.5 g·L^−1^ and the RCDW to 8.2 g·L^−1^. In the same period, the PHB content decreased to 4 wt%. Within this time frame, the *in-line* signals overestimated CDW and RCDW to 13.9 and 12.3 g·L^−1^ at 13 h, respectively. The estimated RCDW was higher than the estimated CDW until 12 h, which yielded a negative calculated PHB content. An accumulation of ammonia up to 195 mM at 19 h was detected, which subsequently decreased and was depleted after 28 h. The CDW and RCDW increased up to 63 and 32 g·L^−1^, respectively, during this period. Contrary to expectation, PHB accumulation was detected from 13 to 19 h up to 33 wt%. Subsequently, the PHB content decreased again to 28 wt% during ammonia consumption and further accumulation started after 25 h. Subsequently, the PHB content increased to 72 wt% at 46 h. The preliminary accumulation and degradation of PHB until 25 h was also detected by the calculated *in-line* PHB signal. Subsequently, the *in-line* PHB content increased to 61 wt% at 46 h and ceased afterwards. At 46 h, the *off-line* CDW and RCDW had increased to 106 and 30 g·L^−1^, respectively. In total, 76 g·L^−1^ PHB accumulated in 46 h, which represents a space time yield of 1.65 g_PHB_·L^−1^·h^−1^ and a yield coefficient of 0.43 g_PHB_·g_Oil_^−1^. The overall urea usage was 0.48 g_Urea_·g_RCDW_^−1^. The *in-line* RCDW signal indicated an accumulation until 29 h to a final RCDW of 29 g·L^−1^. The *in-line* CDW signal increased simultaneously with the *off-line* CDW until 36 h to 82 g·L^−1^. Unexpectedly, the *in-line* CDW signal decreased afterwards until 46 h and stayed constant at a value of 71 g·L^−1^.

The high-cell-density cultivation showed that PDW spectroscopy is capable of a qualitative tracking of the CDW, RCDW, and PHB content. Nevertheless, the quantitative accuracy was not precise during the first 12 h of the cultivation at low cell densities. However, the estimated *in-line* CDW showed a good representation until 36 h. Also, the *in-line* RCDW was estimated from the *µ*_a_ with a very good accuracy for the rest of the cultivation. In contrast, a drop of the *µ*_s_’ signal after 36 h did not correlate with CDW during PHB production.

## 4. Discussion

The purpose of this study was to evaluate the potential of PDW spectroscopy for monitoring plant oil-based *R. eutropha* cultivations. The batch (Figure 2) and pulse-based fed-batch (Figure 3) cultivations showed that the reduced scattering coefficient *µ*_s_’ correlates strongly with the CDW and the absorption coefficient *µ*_a_ with the RCDW (Figure 4). These results demonstrate that PDW spectroscopy is a valuable tool for *in-line* monitoring of the CDW, RCDW, and PHB accumulation. To the best of our knowledge, the results of this study are the first data showing *in-line* quantification of PHB. The lack of such *in-line* or *on-line* monitoring devices for an adaptive control of the production process was recently emphasized by Koller et al. [3]. Previously, Cruz et al. reported the possibility to use a NIR transflectance probe for an *in-line* quantification of PHB. However, the authors showed only *at-line* data quantifying the PHB and CDW concentration (up to 9.3 g·L^−1^ and 13.7 g·L^−1^, respectively) during batch cultivations [34]. 

During the initial batch cultivations, PHB yields of 0.56–0.66 g_PHB_·g_Oil_^−1^ were obtained (Figure 2), which are similar to previously reported yields for *R. eutropha* H16 growth on palm oil [35]. During the high-cell-density fed-batch cultivation 106 g·L^−1^ CDW (72 wt% PHB) with a space time yield of 1.65 g_PHB_·L^−1^·h^−1^ were reached, which is comparable to other published high-cell-density plant oil cultivations [21,24,25,29,36,37]. 

In the pulse experiment, a nitrogen bolus was added (32 h) after the PHB production phase to trigger PHB degradation, as described previously [38]. The PDW spectroscopy signal *µ*_s_‘ decreased with the declining CDW while *µ*_a_ increased with an increasing RCDW (Figure 3). Currently, we do not understand why the strength of the signal was not proportional with the determined *off-line* value changes after that time point (32 h). For this reason, these measurement points were not used for the linear correlation. A potential hypothesis could be an unequal distribution of PHA granules during PHA mobilization, as it was reported for *Pseudomonas putida* [39], which might have an effect on scattering and absorption coefficients during PHA degradation. 

Atypical PHB formation before nitrogen depletion was detected during the high-cell-density cultivation (Figure 5). This preliminary formation of PHB could explain the low yield coefficient of 0.43 g_PHB_·g_Oil_^−1^, which is significant lower than the typical yield of PHB in *R. eutropha* plant oil cultivations [37]. The formation of PHB without nutrient starvation could indicate a stress response triggered by the high urea levels. Stress responses typically involve the formation of the alarmone (p)ppGpp. For *R. eutropha* it is known that formation of this alarmone triggers PHB formation [38,40], but (p)ppGpp formation due to excess urea or ammonia availability was not studied so far. Additionally, it was reported that controlled induction of stress could also been used for an enhanced PHB formation [41]. Such stress responses should be thoroughly considered during the scale-up of a *R. eutropha* PHA production process, as zones of high or low substrate availability occur in large scale bioreactors. The high impact of such substrate gradients on a reduction of biomass and product yields were intensively studied for *Escherichia coli* [42]. Adapting the feeding strategy during the PHA production process by using *in-line* monitoring devices could be a potential scenario for avoiding such negative impacts on the process. 

A reduction of the *µ*_s_’ signal after 35 h was observed during the high-cell-density fed-batch cultivation, whereas *µ*_a_ stayed constant during that period (Figure 5). The decrease of *µ*_s_’ instead of a leveling off of the signal contradicts that a signal saturation effect was observed. Additionally, scattering coefficients in suspensions with particle contents of up to 40% (v·v^−1^) were measured successfully with this technology [9,10]. Heavy foaming, which occurred after 35 h, could be the reason for the observed signal reductions. The surplus of foam was constantly forced into the liquid phase, which increased the overall gas hold-up and total reaction volume in the system. This additional gas volume results in a dilution of the system, which could explain the *µ*_s_’ decrease (35–45 h) even though the culture continued to accumulate PHB (Figure 5). The foaming occurred after the end of the continuous rapeseed oil feed at 35 h. Before 35 h, the added oil functioned as a natural antifoam agent by decreasing the surface tension of the culture broth. During plant oil cultivations foaming occurs through the emulsification process. *R. eutropha* emulsifies plant oils before uptake, which is catalyzed by extracellular lipases. The lipases cleave the triacylglycerols in diacylglycerols, monoacylglycerols, glycerol, and free fatty acids (FFAs) [23,43,44], which causes heavy foaming during aerated bioreactor cultivations. Nevertheless, *µ*_s_’ stayed constant after the CDW did not further increase at 45 h, which could indicate the perfect time point for harvesting in an industrial process. A reliable *in-line* quantification of CDW, RCDW, and subsequently PHA concentration was reached until a CDW of 84 g·L^−1^. To increase the robustness of the method further at higher cell densities, the calculated gas-hold up in the bioreactor [45] could be integrated in the correlation of the PDW spectroscopy signals. In order to quantify the direct impact of the PHA concentration on the optical coefficients, further studies referencing cell counts and sizes by flow cytometry and microscopy need to be conducted.

A wavelength of 638 nm was used to evaluate the PDW spectroscopy signals during this study, which did not show any correlations with the oil addition or emulsification (Figure 3). The emulsification process is very important for an efficient growth. It was previously shown that an overexpression of lipases results in a reduced lag phase and subsequently a more efficient process [43]. In previous studies, it was shown that PDW spectroscopy was used to measure emulsions [46,47]. An *in-line* determination of the oil content or the emulsion formation would be a very valuable additional information for bioprocess development and process control. This information might result from integration of additional wavelengths into the PDW spectroscopy set-up. 

To summarize, PDW spectroscopy allows *in-line* estimation of the CDW, RCDW, and PHB content in real-time. In contrast, *off-line* analysis is typically carried out to determine the PHB content, which includes drying the cells and polymer derivatization before time-consuming HPLC or GC analysis. By using PDW spectroscopy, process development and scale-up could be accelerated. In addition, this technology could be used at a large scale for process monitoring and control of *R. eutropha* cultivations. Specifically, the real-time adjustment of feeding strategies according to the PHA production rates—determined by PDW spectroscopy signals—holds great potential. 

## 5. Conclusions

Here, we show that *in-line* PDW spectroscopy is a powerful PAT tool for monitoring *R. eutropha*-based PHB production. The reduced scattering coefficient *µ*_s_’ and absorption coefficient *µ*_a_ showed very reproducible signals during different biological cultivations. The new method described in this study allows *in-line* monitoring of CDW, RCDW, and PHB concentrations in *R. eutropha* cultivations up to a CDW of 84 g·L^−1^. PDW spectroscopy could contribute to improving the scaling-up process and thus to performing PHA production processes in an economical efficient way with the ultimate goal to commercialize a green sustainable plastic. 

## Supplementary Materials

The following are available online at https://www.mdpi.com/2306-5354/6/3/85/s1, Figure S1: Detailed figure of Batch 1, Figure S2: Detailed figure of Batch 2, Figure S3: Detailed figure of Batch 3, Figure S4: Ammonia contents from the pulse feed cultivations.

## Appendix A

CDW [g·L^−1^] = 19.012 [mm·g·L^−1^] µ_s_’ [mm^−1^] − 1.2404 [g·L^−1^] R^2^ = 0.96 (A1)

RCDW [g·L^−1^] = 1872 [mm·g·L^−1^] µ_a_ [mm^−1^] + 2.5722 [g·L^−1^] R^2^ = 0.90 (A2)

PHB [wt%] = 100 [wt%] (CDW [g·L^−1^] – RCDW [g·L^−1^])/CDW [g·L^−1^] (A3)
