# Supplementary material for: In-Line Monitoring of Polyhydroxyalkanoate (PHA) Production during High-Cell-Density Plant Oil Cultivations Using Photon Density Wave Spectroscopy
Source: Bioengineering (Basel). 2019 Sep 19;6(3):85. doi: 10.3390/bioengineering6030085 (PMC6783927; doi:10.3390/bioengineering6030085)
Supplement: Supplementary file 1 [file bioengineering-06-00085-s001.pdf]

bioengineering

Supplementary Materials

Article

In-Line Monitoring of Polyhydroxyalkanoate (PHA) Production during High-Cell-Density Plant Oil Cultivations Using Photon Density Wave Spectroscopy

Björn Gutschmann [1], Thomas Schiewe [2], Manon T. H. Weiske [1], Peter Neubauer [1], Roland Hass [2], Sebastian L. Riedel [1,] *

[1] Bioprocess Engineering, Department of Biotechnology, Technische Universität Berlin, Ackerstraße 76, 13355 Berlin, Germany

[2] innoFSPEC, University of Potsdam, Am Mühlenberg 3, 14476 Potsdam, Germany

* Correspondence: riedel@tu-berlin.de

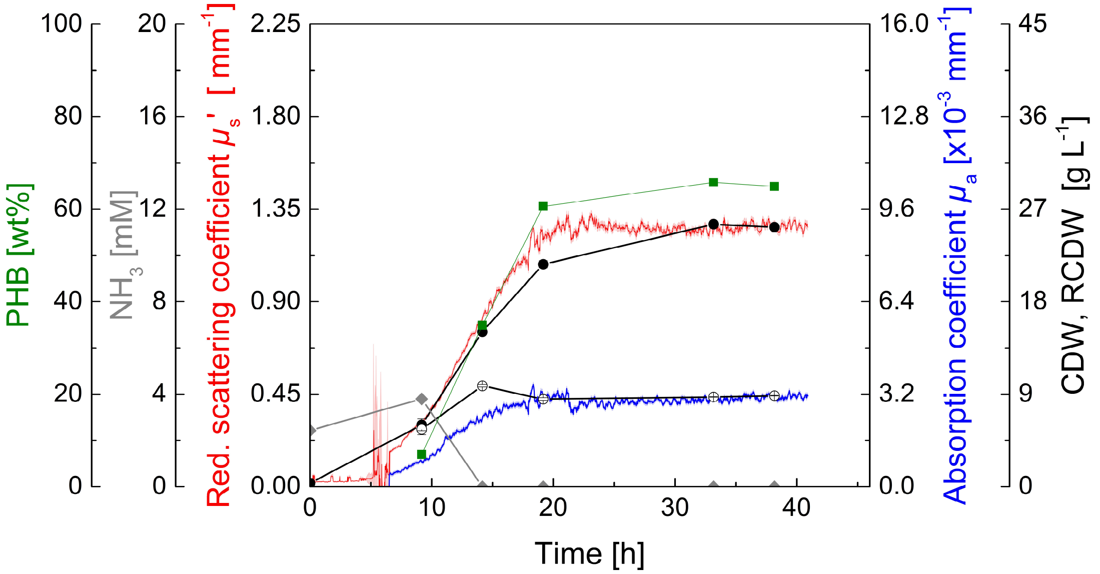

Figure S1. Batch 1, cultivation for PHB production by *R. eutropha* H16 using 3% (w v^{-1}) rapeseed oil and 2.25 g L^{-1} urea as the carbon and nitrogen sources, respectively. Ammonia content (grey diamonds, mM), PHB content (green squares, wt%), CDW (filled circles, g L^{-1}), RCDW (empty circles, g L^{-1}), reduced scattering coefficient μ_s' (red line, mm^{-1}) and absorption coefficient μ_a (blue line, x10^{-3} mm^{-1}) are shown.

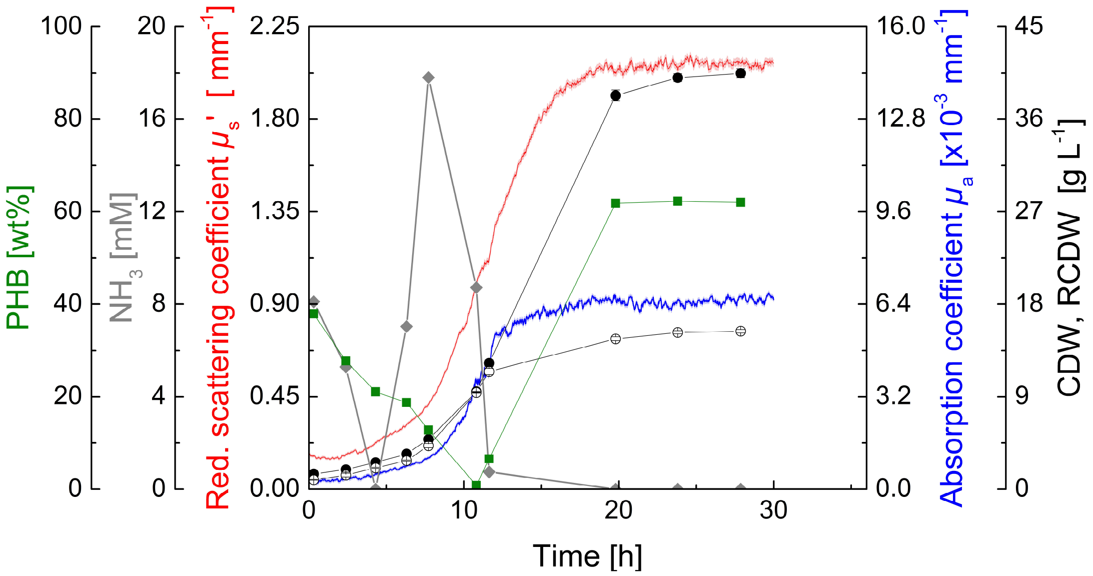

Figure S2. Batch 2, cultivation for PHB production by *R. eutropha* H16 using 4% (w v^{-1}) rapeseed oil and 4.5 g L^{-1} urea as the carbon and nitrogen sources, respectively. Ammonia content (grey diamonds, mM), PHB content (green squares, wt%), CDW (filled circles, g L^{-1}), RCDW (empty circles, g L^{-1}), reduced scattering coefficient μ_s' (red line, mm^{-1}) and absorption coefficient μ_a (blue line, $x10^{-3}$ mm^{-1}) are shown.

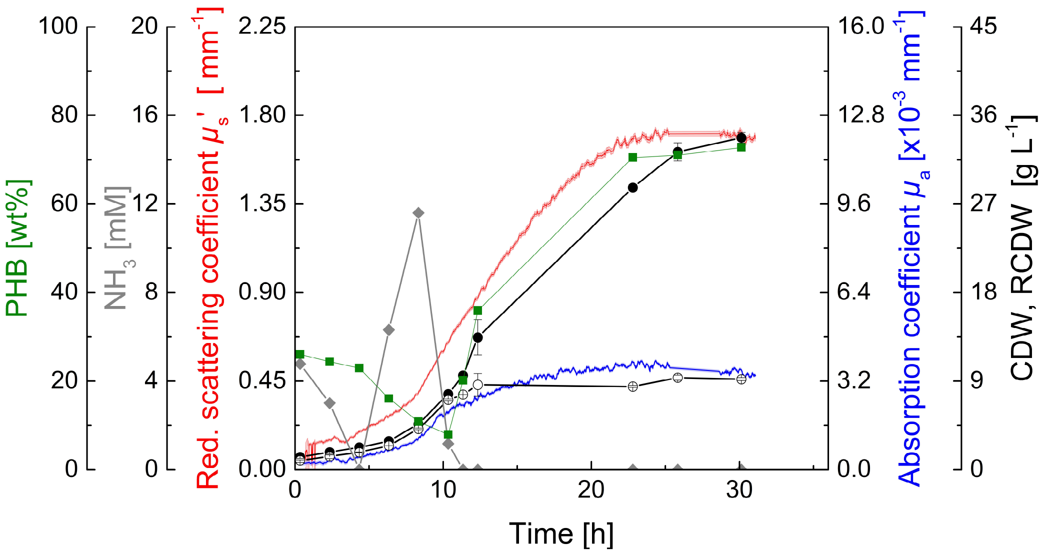

Figure S3. Batch 3, cultivation for PHB production by *R. eutropha* H16 using 4% (w v^{-1}) rapeseed oil and 2.25 g L^{-1} urea as the carbon and nitrogen sources, respectively. Ammonia content (grey diamonds, mM), PHB content (green squares, wt%), CDW (filled circles, g L^{-1}), RCDW (empty circles, g L^{-1}), reduced scattering coefficient μ_s' (red line, mm^{-1}) and absorption coefficient μ_a (blue line, $x10^{-3}$ mm^{-1}) are shown.

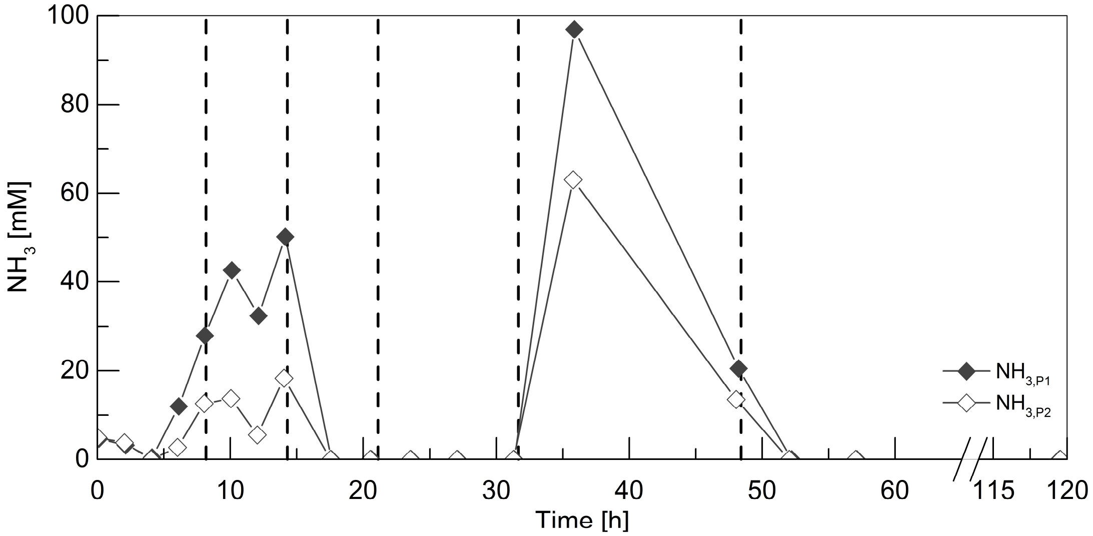

Figure S4. Ammonia contents from the pulse feed cultivations. The cultures were initially started with 0.5% (w v^{-1}) rapeseed oil and 150 mM nitrogen (4.5 g L^{-1} urea). The dashed vertical lines represent time points of pulse addition: 0.5% (w v^{-1}) rapeseed oil at 8.2 h, 1% (w v^{-1}) rapeseed oil at 14.3 h, 2% (w v^{-1}) rapeseed oil at 21.1 h, 0.5% (w v^{-1}) rapeseed oil at 8.2 h, a bolus (110 mL urea solution (122 g L^{-1}), 15.6 mL 0.5 M K_2SO_4, 30 mL 0.32 M $MgSO_4$, 30 mL 0.042 mM $CaCl_2$ and 3 mL trace element solution) at 31.7 h and 4% (w v^{-1}) rapeseed oil at 48.4 h. Ammonia contents from the reference experiment (dark grey, filled diamonds, mM) and the biological duplicate (dark grey, open diamonds, mM) are shown.
